# SG-APSIC1199: Evaluation of a pooling strategy using Xpert Carba-R assay for screening for carbapenemase-producing organisms in rectal swabs

**DOI:** 10.1017/ash.2023.79

**Published:** 2023-03-16

**Authors:** Peng Zhang, Qi Wang, Chaoe Zhou, Xinghui Gao, Yi-wei Tang, Hui Wang

**Affiliations:** Peking University People’s Hospital, Beijing, China; Peking University People’s Hospital, Beijing, China; Peking University People Beijing’s Hospital, Beijing, China; Danaher Diagnostic Platform China/Cepheid, Shanghai, China; Danaher Diagnostic Platform China/Cepheid, Shanghai, China; Peking University People’s Hospital, Beijing, China

## Abstract

**Objectives:** Rapid and accurate screening for carbapenemase-producing organism (CPOs) in hospitalized patients is critical for infection control and prevention. The Xpert Carba-R assay is designed for rapid detection of CPOs, but 1 assay is usually conducted for only 1 sample. We evaluated a pooling strategy for CPO screening using the Xpert Carba-R assay. **Methods:** Swab sets containing 2 swabs were collected from 415 unique patients at Peking University People’s Hospital. One swab was used for the pooling test, in which 5 swabs from different patients were mixed in 1 sample treatment solution. The prevalence of CPOs in the hospital (5.3%) predicted that 5:1 pooling was most economical. As the reference method, the other swab was tested by culture using sequencing. **Results:** Of 415 samples, 383 were CPO negative using the pooling test strategy and 31 were positive. All samples that were negative by pooling were negative by culture and sequencing. Among the 31 positive samples identified by the pooling strategy, 26 were positive by culture and sequencing (including 24 samples with 1 targeted gene and 2 samples with double targeted genes, 1 NDM+/IMP+ and 1 VIM+/IMP+), and 5 were negative. Overall, 198 tests were conducted in the study, and 217 were saved compared with testing individually. The efficiency of the pooling strategy was 215%. The overall sensitivity was 1 (95% CI, 0.840–1), the specificity was 0.987 (95% CI, 0.968–0.995), the accuracy was 0.987 (95% CI, 0.970–0.996), positive predictive value was 0.838 (95% CI, 0.655–0.939), and the negative predictive value was 1 (95% CI, 0.988–1). **Conclusions:** The pooling strategy using the Xpert Carba-R assay showed good potential in screening CPO with good sensitivity and a significantly lower cost.

